# Prospective, Open-Label, Observational, Multicenter, Single Arm, Post-Marketing Study in Asthmatic Patients for Evaluation of Safety and Effectiveness of Indacaterol/Mometasone DPI (PROMISING-SHIFT)

**DOI:** 10.3390/arm93010003

**Published:** 2025-02-06

**Authors:** Saurabh Karmakar, Gajendra V. Singh, Amit S. Bhate, Vijaykumar Barge, Bharat Mehrotra, Chintan Patel, Ekta Sinha, Sagar Bhagat, Saiprasad Patil, Hanmant Barkate

**Affiliations:** 1 Department of Pulmonary Medicine, All India Institute of Medical Sciences, Patna 801507, India; drkarmakar01@gmail.com; 2 Department of Pulmonary Medicine, Sarojini Naidu Medical College, Agra 282003, India; drsinghgv@grnail.com; 3 Department of Pulmonary Medicine, Jeevan Rekha Hospital, Belagavi 590010, India; dr.amitsureshbhate@grnail.com; 4 Department of Medicine, Chhatrapati Pramilaraje Hospital, Kolhapur 416012, India; drvijaybarge12@gmail.com; 5 Department of Pulmonary Medicine, New Leelamani Hospital Pvt Ltd., Kanpur 208001, India; bharatmehrotracr@gmail.com; 6 Department of Pulmonary Medicine, Aatman Hospital, Ahmedabad 380058, India; cr.aatman@gmail.com; 7 Global Medical Affairs, Glenmark Pharmaceutical Limited, Mumbai 400099, India; sagar.bhagat@glenmarkpharma.com (S.B.); saiprasad.patil@glenmarkpharma.com (S.P.); hanmant.barkate@glenmarkpharma.com (H.B.)

**Keywords:** asthma, Indacaterol/Mometasone, LABA, inhaled corticosteroids

## Abstract

**Highlights:**

**What are the main findings?**

- The once-daily Indacaterol/Mometasone (IND/MF) DPI demonstrated a favorable safety profile over 12 weeks, with adverse events (AEs) reported in only 14.37% of patients, which were all mild to moderate in nature and not serious adverse events (SAEs);
- Significant improvements in lung function (trough FEV1 and FVC, *p* < 0.001), asthma control (ACQ-5, *p* < 0.001), reduced exacerbations, and lower rescue medication use were observed, and a majority of the patients and physicians were satisfied with the treatment.

**What does the primary finding suggest?**

- The absence of serious adverse events and the mild nature of reported AEs confirm the safety and tolerability of IND/MF DPI, making it suitable for long-term use in asthma management;
- IND/MF DPI can be a promising alternative for asthma management in patients inadequately managed with prior treatments, offering improved lung function, fewer exacerbations, and enhanced adherence, contributing to better disease management and enhanced quality of life.

**Abstract:**

Background: Asthma significantly impacts global health, necessitating effective management strategies. A combination of inhaled corticosteroids (ICSs) and long-acting β2-agonists (LABA) is recommended for patients with inadequately controlled asthma. Method: This prospective, open-label, multicenter study (PROMISING-SHIFT) study evaluated the safety and efficacy of once-daily Indacaterol/Mometasone (IND/MF) dry powder inhaler (DPI) in Indian asthma patients (≥12 years), inadequately controlled with prior therapies. Patients received IND/MF DPI in three strengths (150/80 mcg, 150/160 mcg, 150/320 mcg) over 12 weeks. Results: The study included a total of 174 participants, and 27 adverse events (AEs) in 25 patients (14.37%) were reported, primarily mild to moderate, with no serious adverse events (SAEs). Drug-related treatment-emergent adverse events (TEAEs) were observed in 11 patients. Significant improvements were noted in the mean trough FEV1 and FVC, increasing from baseline to week 4 and week 12 (*p* < 0.001). The mean ACQ-5 score significantly decreased from 3.0 ± 0.73 baseline to 2.50 ± 0.53 (16.67%) at week 4 and further to 1.73 ± 0.35 at week 12, along with reduced exacerbations (*p* < 0.001). The need for rescue medication declined from 13.79% to 8.62%, and 96.55% of patients reported treatment satisfaction by study completion. Conclusion: Once-daily IND/MF DPI demonstrated a favorable safety profile with marked improvements in lung function, asthma control, and patient satisfaction, making it a promising option for long-term asthma management in Indian patients.

## 1. Introduction

Asthma is a chronic respiratory condition characterized by variable airflow obstruction, bronchial hyperresponsiveness, and airway inflammation [1], affecting 262 million people worldwide [2]. The substantial burden of asthma leads to significant morbidity, a diminished quality of life, and high healthcare costs [3]. According to the recent Global Burden of Disease (GBD, 1990–2019) report, asthma affects 34.3 million people in India, representing 13.09% of the global burden [4].

International guidelines, including those from the Global Initiative for Asthma (GINA 2024), recommend a stepwise approach to management, emphasizing long-acting beta-2 agonist (LABA) and inhaled corticosteroid (ICS) combinations for patients at GINA step 3 and above, particularly adolescents and adults aged 12 and older [5]. The joint recommendations from the Indian Chest Society (ICS) and the National College of Chest Physicians (NCCP) of India in the 2015 guidelines also advise that when asthma symptoms remain uncontrolled despite moderate doses of ICS monotherapy, adding a LABA to the treatment regimen is preferred [6]. The Indian Medical Association (2020) and Indian Academy of Pediatrics (IAP) 2022 guidelines also recommend low/medium-dose ICS plus LABA, as needed or daily, for asthma management (step 3 and above) in patients aged 12 years and older [7,8]. LABAs are combined with ICS to provide prolonged bronchodilation, improving airflow and reducing bronchoconstriction [9].

Despite the benefits of conventional LABA/ICS combinations, non-adherence remains a challenge due to complex regimens, inhaler technique issues, and misconceptions regarding controller medications [10]. In this context, once-daily LABA/ICS formulations have shown improved efficacy, adherence, and asthma control compared to twice-daily regimens [11]. The ICS mometasone furoate (MF) and the ultra LABA indacaterol acetate (IND) have been formulated as FDC therapy IND/MF delivered via DPI one time a day. IND is a potent beta-2 adrenoceptor agonist with a rapid onset of action (7.8 min) which is comparable to formoterol and salbutamol, but significantly faster than salmeterol (19.4 min), as demonstrated in a study on isolated human bronchi [12]. Combined with MF, it enhances lung function while maintaining a strong safety profile and minimal systemic steroid effects due to low bioavailability [10]. The DPI is available in three strengths, IND/MF: 150/80 mcg, 150/160 mcg, and 150/320 mcg, providing physicians with flexibility to adjust ICS doses according to asthma severity as recommended by the GINA guidelines.

This post-marketing surveillance (PMS) study evaluates the safety and effectiveness of once-daily IND/MF DPI in the Indian population, providing insights towards its use in clinical practice.

## 2. Material and Methods

### 2.1. Study Design

This prospective, open-label, multicenter, single-arm, post-marketing study assessed the safety and effectiveness of Indacaterol/Mometasone DPI in asthmatic patients aged 12 years and above, diagnosed per GINA 2022 guidelines. The study was conducted across six sites in India and adhered to the protocol, New Drugs and Clinical Trials Rules 2019 (India), the ethical principles outlined in the Declaration of Helsinki (64th WMA General Assembly, 2013), ICH Good Clinical Practice (GCP) guidelines, and all applicable local regulations. Ethics committee approval was obtained from each participating site before initiation. The study was listed with the Clinical Trials Registry of India (CTRI/2023/05/052297).

### 2.2. Study Participants

Individuals aged 12 years and above with a confirmed diagnosis of asthma, and who were prescribed Indacaterol/Mometasone DPI, were eligible for the study if they had a pre-bronchodilator FEV1 of 40–90% predicted normal and were symptomatic while receiving ongoing treatment with ICS-SABA, ICS-LABA, or MART (general maintenance and reliever therapy, BUD/FOR and MF/FOR) therapy. An ACQ-5 score of ≥1.5 at baseline was required. Eligible participants had to provide written informed consent/parental consent, or permission and be willing to adhere strictly to the investigator’s prescription. Individuals were excluded if they had been recently hospitalized for a life-threatening condition or acute asthma exacerbation, smoked more than ten pack-years, or had participated in another clinical trial within 30 days before enrollment. Women of childbearing potential were not restricted but required a risk–benefit assessment by the investigator before starting treatment. Patients with known hypersensitivity to any study drug or excipient were excluded.

### 2.3. Study Product

Patients aged ≥12 years with asthma, previously treated with ICS/SABA, ICS/LABA, or MART (general maintenance and reliever therapy, BUD/FOR and MF/FOR), were enrolled in the study and assigned Indactiv^®^ DPI. The investigational product included Indacaterol 150 mcg combined with Mometasone Furoate 80 mcg, 160 mcg, or 320 mcg powder for inhalation, administered once daily via a Dry Powder Inhaler. Doses were selected based on routine clinical practice and administered at the clinic under supervision. Only SABA was permitted as rescue medication, while other systemic corticosteroids, methylxanthines, beta-blockers, diuretics, inhaled maintenance drugs, and monoclonal antibodies were prohibited. Treatment compliance, defined as at least 80% dosing adherence, was monitored by study personnel through medication counts and patient diaries, with compliance reviewed by clinical research associates during site visits.

### 2.4. Outcome Measures

The primary objective of the study is to evaluate the safety of Indacaterol/Mometasone DPI in asthma management. Safety was assessed by monitoring treatment-emergent adverse events (TEAEs) (any event not present prior to the initiation of treatment or any event already present and that worsens in either intensity or frequency following exposure to the drug treatment), drug-related TEAEs (any TEAE with at least a possible relationship to the study treatment as assessed by the investigator), and serious adverse events (SAEs) (any untoward medical occurrence at any dose which is life-threatening requiring hospitalization or death and necessitates discontinuation of study drug at the discretion of the treating physician) over a 12-week period. Efficacy was measured by changes in trough FEV1, ACQ-5 scores, exacerbation rates, rescue medication use, and patient and physician satisfaction with the treatment.

### 2.5. Statistical Analysis

The biostatistician conducted data analysis using a finalized statistical analysis plan (SAP). All statistical procedures were carried out using R Software version 4.3.2 Continuous endpoints were summarized with descriptive statistics, such as the number of patients, mean, standard deviation, minimum, median, and maximum, while categorical endpoints were presented with frequency and percentage.

The study’s primary objective was to evaluate the safety of IND/MF DPI. Sample size determination was calculated, taking into consideration the post hoc analysis from landmark trials, which had observed 14% and 11.5% of patients with any drug-related TEAEs receiving high-dose IND/MF and medium dose IND/MF, respectively. For 12-week treatment with low, medium, and high-dose IND/MF, the rate of drug-related TEAEs was assumed to be 13%. A single-group design was used to obtain a two-sided 95% confidence interval for a single proportion. The sample proportion was estimated to be 0.13. In order to achieve a confidence interval with a width not exceeding 0.1, a total of 174 participants were required. The sample size was computed using PASS 2022, version 22.0.2.

## 3. Results

**Figure 1:**
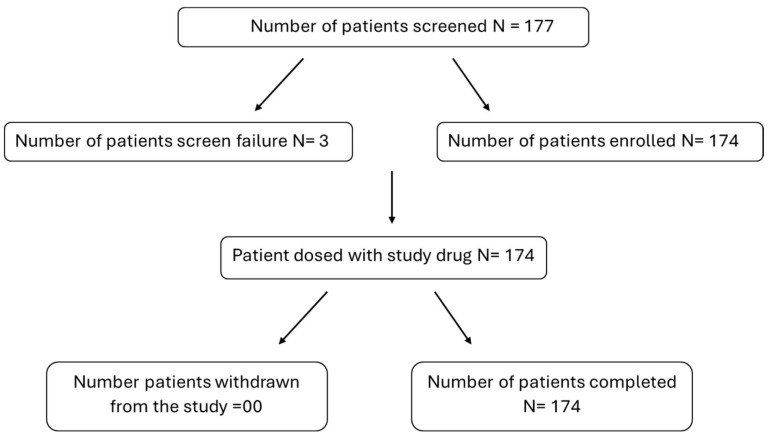
Flowchart of patients in the study.

A total of 177 subjects were screened for the study, out of which 174 subjects were enrolled in the study; 3 subjects were screened and failed, with 2 subjects not meeting the inclusion criteria in terms of pre-bronchodilator FEV1 value, while 1 subject was simultaneously involved in another clinical study (Figure 1).

We enrolled 174 patients with a mean age of 33.31 ± 14.89 years comprising 71 (40.80%) female and 103 (59.19%) male patients. Allergic rhinitis was the most common comorbidity occurring in 9% of patients, followed by dyslipidemia and hypertension. Regarding prior medication for asthma, 68.39% had received ICS-LABA. Patient characteristics are presented in Table 1. Notably, all 174 patients (100%) adhered to their treatment regimens, demonstrating full treatment compliance throughout the study.

## 4. Safety and Effectiveness Evaluation

All 174 patients successfully completed the 12-week treatment duration, of which, 34 (19.5%) patients were put on low dose IND/MF (150/80 mcg), 92 (53%) patients on IND/MF (150/160 mcg), and 48 (27.5%) patients were on IND/MF (150/320 mcg). A total of 25 patients (14.37%) experienced 27 adverse events (AEs), as summarized in Table 2. Among these events, 18 (66.6%) were classified as mild, and 9 (33.3%) as moderate. Additionally, 11 patients (6.32%) experienced 11 drug-related treatment-emergent adverse events (TEAEs), also detailed in Table 2. No serious adverse events (SAEs) were reported during the study, and all cases were fully resolved with appropriate medication.

## 5. Effectiveness Analysis

**Figure 2:**
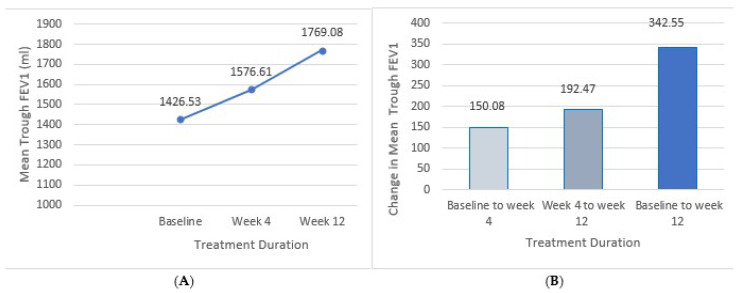
Improvements in mean trough FEV1 throughout the 12-week treatment period. (**A**) Mean trough FEV1 at baseline; week 4 and week 12 of treatment. (**B**) Mean change in trough FEV1 from baseline to week 4, week 4 to week 12, and from baseline to week 12.

The mean trough FEV1 increased from 1426.53 ± 406.26 mL at baseline to 1576.61± 518.78 mL at week 4 and 1769.08 ± 543.02 mL at week 12, respectively. The mean change in trough FEV1 from baseline was 150.08 ± 112.52 mL and 342.55 ± 136.26 mL at week 4 and week 12, respectively, both of which were statistically significant (*p* < 0.0001). These results are illustrated in Figure 2.

**Figure 3:**
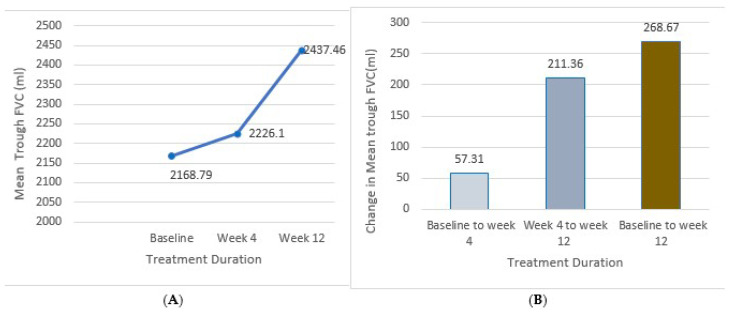
Improvements in mean trough FVC throughout the 12-week treatment period. (**A**) Mean trough FVC at baseline; week 4 and week 12 of treatment. (**B**) Mean change in trough FVC from baseline to week 4, week 4 to week 12, and from baseline to week 12.

The mean trough FVC (mL) of the patients increased from 2168.79 ± 777.46 at baseline to 2226.1 ± 705.61 at week 4 and further to 2437.46 ± 720.50 at week 12. The mean change in trough FVC from baseline was 57.31 ± 71.85 mL at week 4 and 268.67 ± 56.96 mL at week 12. Both improvements were statistically significant (*p* < 0.0001). These results are illustrated in Figure 3.

**Figure 4:**
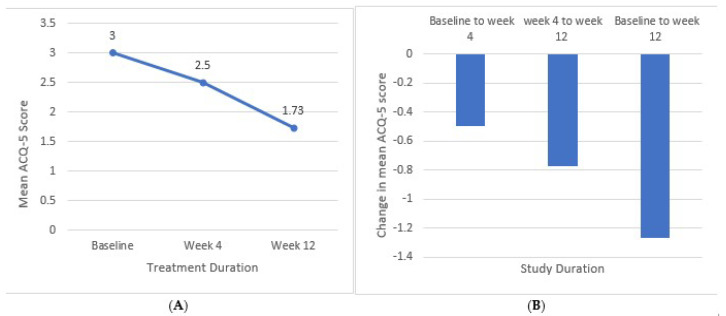
Reduction in mean ACQ-5 scores during the treatment period. (**A**) Mean ACQ-5 score at baseline, week 4, and week 12 of treatment. (**B**) Change in mean ACQ-5 score from baseline to week 4, week 4 to week 12, and from baseline to week 12.

Compared to the baseline, a notable decrease in the ACQ-5 score was observed at week 4 and week 12. The mean ACQ-5 score decreased from 3.0 ± 0.73 at baseline to 2.50 ± 0.53 (16.67%) at week 4 and further to 1.73 ± 0.35 (42.33%) at week 12. The mean change from baseline was −0.5 ± 0.20 at week 4 and −1.27 ± 0.38 at week 12, with both changes being statistically significant (*p* < 0.001), as illustrated in Figure 4.

**Figure 5:**
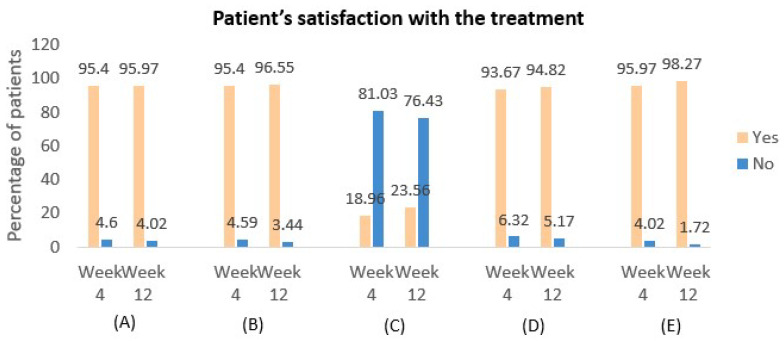
Patient satisfaction with asthma treatment over 12 weeks. (**A**) Do you find it easy to fit your asthma medication into your everyday life? (**B**) When you take your medication, do you feel confident that your asthma symptoms will be controlled? (**C**) Do you worry that you are not taking the right medication for your asthma symptoms? (**D**) Do you find it easy to carry your asthma inhaler(s) with you, and do not have any taste or smell complaints? (**E**). Do you feel confident in using your inhaler(s)?

**Figure 6:**
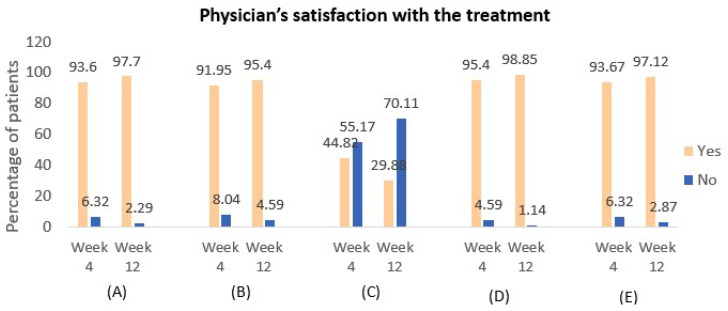
Physician’s satisfaction with asthma treatment over 12 weeks. (**A**) Do you feel that patient’s asthma medication fits into their everyday life? (**B**) Do you feel confident in the ability of this medication to control patient’s asthma symptoms? (**C**) Do you have any worries or uncertainties about whether this is the appropriate medication for this patient? (**D**) Do you feel it is convenient for the patient to carry their asthma inhaler with them and not have any taste or smell complaints? (**E**) Does the patient demonstrate confidence in their ability to use their inhaler(s) effectively?

A total of 22 patients (12.64%) experienced mild exacerbations at week 4, which decreased to 13 patients (7.47%) by week 12. Notably, no moderate or severe exacerbations were reported during the study. Additionally, the use of rescue medication with salbutamol was observed in 13.79% of patients from week 0 to week 4, and this decreased to 8.62% from week 4 to week 12, reflecting a reduced need for additional asthma control as treatment progressed. The study further assessed patient satisfaction with the treatment, revealing that 166 patients (95.40%) at week 4 and 168 patients (96.55%) at week 12 expressed confidence in their medication’s ability to control asthma symptoms (Figure 5). Physician satisfaction with the treatment was also evaluated, with findings presented in Figure 6.

## 6. Discussion

In this post-marketing surveillance study involving Indian patients with inadequately controlled asthma despite prior treatment, the objective was to evaluate the safety and effectiveness of once-daily IND/MF at three different dose strengths: 150/80 mcg, 150/160 mcg, and 150/320 mcg. The safety data from this PMS study indicate a well-tolerated profile for the once-daily inhaler, demonstrating its suitability for long-term asthma management.

The findings from two pivotal phase III studies, PALLADIUM and IRIDIUM, provide robust evidence regarding the efficacy and safety of high- and medium-dose IND/MF. These studies revealed that the IND/MF combination not only effectively controlled asthma symptoms but also exhibited a favorable tolerability profile. This evidence supports the inclusion of IND/MF as a viable option for long-term asthma management, reinforcing its potential to enhance treatment outcomes in patients struggling with asthma control [11].

In this study, a small percentage of patients experienced adverse events (AEs), most of which were mild and effectively managed with appropriate treatment. The most reported AEs were cough, upper respiratory tract infections (URTIs), and fever, with only a few cases attributed to the treatment. Importantly, no serious adverse events (SAEs) were observed. These results can be compared with a post hoc analysis of pooled data from the PALLADIUM and IRIDIUM studies, where the incidence of SAEs reported were 6.9% in patients with high-dose IND/MF. In the study, asthma exacerbations were the most frequently reported AEs and SAEs that resulted in treatment discontinuation across the treatment groups in 1.4% of the study population on high-dose IND/MF. Notably, there was no increased risk of adverse events in the high-dose group compared to the medium-dose group, and our study similarly did not raise any additional safety concerns leading to treatment discontinuation [13]. These findings further confirm the well-tolerated profile of the once-daily IND/MF dry powder inhaler.

The PALLADIUM trial by van Zyl-Smit RN et al. (2020) showed that both high-dose and medium-dose IND/MF significantly enhanced trough FEV1 from baseline to week 26 (*p* < 0.001). Furthermore, high-dose IND/MF demonstrated comparable trough FEV1 improvement at week 26 and significant improvement at week 52 as compared to high-dose SAL/FLU [14]. Our study aligns with these findings, demonstrating similar robust improvements in lung function over a 12-week period, with efficacy evident as early as 4 weeks. The Phase III QUARTZ study, which evaluated low-dose IND/MF, further supports these findings, showing significant benefits for lung function [15]. These consistent results across studies underscore the effectiveness of IND/MF in improving asthma control, with our study confirming its efficacy in a shorter, 12-week timeframe.

This study showed significant improvement in asthma control, as evidenced by the ACQ-5 scores, which showed a notable reduction by week 4 and further improvement by week 12 compared to baseline. This aligns with findings from Chapman K et al., who reported similar decreases in ACQ-7 scores with various doses of IND/MF [11]. These results indicate that the IND/MF combination effectively enhances asthma control, with substantial improvements seen as early as 4 weeks and sustained through 12 weeks of treatment.

In our study, moderate or severe asthma exacerbations were remarkably low, with only a small percentage of patients experiencing mild exacerbations. This is in contrast to other studies, such as those by Chapman et al. (2021) and Van Zyl-Smit RN et al. (2020), which reported moderate and severe exacerbations but demonstrated a significant reduction in these events with high-dose IND/MF once daily compared to other treatment regimens [11]. Similar depletion in moderate or severe, severe, and all exacerbations were also noted in a study conducted by Van Zyl-Smit RN et al., 2023 [16]. The low incidence and severity of exacerbations in our study also correlated with a decrease in the use of rescue medications over the 12-week period. These findings align with larger trials like PALLADIUM and IRIDIUM, which also observed a greater number of rescue medication-free days in patients using high-dose IND/MF once daily (Δ 3.2%, 95% CI 0.1% to 6.3%, *p* = 0.044) [14,16] suggesting that the once-daily IND/MF DPI not only effectively controls asthma symptoms but also minimizes the need for additional medications, thereby improving overall patient management.

Furthermore, this study included additional efficacy measures not previously reported, such as patient and physician satisfaction with the treatment. The findings demonstrated high levels of satisfaction among both patients and physicians, with most patients finding it easy to incorporate asthma medication into their daily routines and expressing confidence in their ability to manage asthma symptoms with the prescribed inhaler. Similarly, the majority of physicians reported confidence in the treatment’s effectiveness and ease of use for their patients. Notably, concerns about taking the right medication decreased over time, while satisfaction with the portability and sensory experience of the inhaler remained consistently high. These outcomes underscore the user-friendly nature of the Indacaterol/Mometasone DPI and its positive impact on both patient adherence and overall treatment experience.

There were a few limitations associated with the current study. It was only possible to measure the improvement in lung function before and after using IND/MF since it was an open-label design and lacked a control group. Also, the duration of this study was only 12 weeks, which may not have been adequate to assess the impact of IND/MF on exacerbation. Additionally, adverse events were collected based on patient’s self-reported data as it was an observational study.

## 7. Conclusions

Indacaterol/Mometasone DPI was found to be well tolerated and effective in managing asthma. This once-daily DPI significantly improved lung function, reduced exacerbations, decreased the need for rescue medications, and increased patient satisfaction. These results suggest that Indacaterol/Mometasone DPI represents a promising shift in asthma treatment, offering substantial benefits over existing therapies.

## Figures and Tables

**Table 1:** Baseline characteristics of study population.

Parameter	
Age (mean ± SD)	33.31 ± 14.89 years
Gender—*n*(%) Male Female	103 (59.19%)
	71 (40.80%)
Baseline % FEV1	58.4± 10.6%
Baseline FEV1 (mean ± SD)	1426.53 ± 406.26 mL
Baseline FVC (mean ± SD)	2168.79 ± 777.46 mL
Baseline ACQ 5 Score (mean ± SD)	3.0 ± 0.73
Exacerbation at baseline (*n*)	0
Comorbidities—n *n*(%)	
Allergic Rhinitis	16 (9.19%)
Dyslipidemia	12 (6.89%)
Hypertension	7 (4.02%)
T2DM	5 (2.87%)
Cardiovascular disease	2 (1.14%)
Prior medication for asthma—*n* (%)	
**ICS-LABA**	**119 (68.39%)**
MART	
-Budesonide + Formoterol	40 (22.98%)
-Mometasone + Formoterol	21 (12.06%)
Fluticasone + Formoterol	57 (32.75%)
Fluticasone + Salmeterol	1 (0.57%)
**ICS-SABA**	**55 (31.60%)**
Salbutamol + Budesonide	45 (25.86%)
Salbutamol + Beclomethasone	7 (4.02%)
Salbutamol + Fluticasone	3 (1.72%)

ICS: Inhaled corticosteroid; LABA: long-acting beta-agonist; SABA: short-acting beta-agonist; FEV1: Forced expirotory volume in 1 sec; FVC: Forced vital capacity; ACQ: Asthma control questionnaire.

**Table 2:** Summary of adverse events.

Adverse Event	No. of AE Reported (*n* %)
**Treatment-emergent adverse effects (TEAEs)**	
**Overall**	27 (14.36%)
Cough	8 (4.59%)
URTI	6 (3.44%)
Fever	3 (1.72%)
Headache	2 (1.14%)
Rhinitis	2 (1.14%)
Body pain	2 (1.14%)
Bronchitis	1 (0.57%)
Runny nose	1 (0.57%)
Sore throat	1 (0.57%)
Stuffy nose	1 (0.57%)
**Drug-related TEAEs**	
**Overall**	11(6.32%)
Cough	5 (2.87%)
URTI	4 (2.29%)
Rhinitis	1 (0.57%)
Bronchitis	1 (0.57%)

*n* (%): Percentage of patients with AE; URTI: Upper respiratory tract infections; TEAEs: Treatment emergent adverse events.

## Data Availability

The data presented in this study are available on request from the corresponding author.
